# Dynamics of the Interaction Between Ceria and Platinum During Redox Processes

**DOI:** 10.3389/fchem.2019.00057

**Published:** 2019-02-08

**Authors:** Paola Luches, Gabriele Gasperi, Marc Sauerbrey, Sergio Valeri, Jens Falta, Jan Ingo Flege

**Affiliations:** ^1^Istituto Nanoscienze, Consiglio Nazionale delle Ricerche, Modena, Italy; ^2^Dipartimento di Scienze Fisiche, Informatiche e Matematiche, Università degli Studi di Modena e Reggio Emilia, Modena, Italy; ^3^Institute of Solid State Physics, University of Bremen, Bremen, Germany; ^4^MAPEX Center for Materials and Processes, University of Bremen, Bremen, Germany; ^5^Applied Physics and Semiconductor Spectroscopy, Brandenburg University of Technology Cottbus-Senftenberg, Cottbus, Germany

**Keywords:** cerium oxide, platinum, LEEM, LEED, reduction

## Abstract

The work is focused on understanding the dynamics of the processes which occur at the interface between ceria and platinum during redox processes, by investigating an inverse catalytic model system made of ceria epitaxial islands and ultrathin films supported on Pt(111). The evolution of the morphology, structure and electronic properties is analyzed in real-time during reduction and oxidation, using low-energy electron microscopy and spatially resolved low-energy electron diffraction. The reduction is induced using different methods, namely thermal treatments in ultra-high vacuum and in H_2_ as well as deposition of Ce on the oxide surface, while re-oxidation is obtained by exposure to oxygen at elevated temperature. The use of two different epitaxial systems, continuous films and nanostructures, allows determining the influence of platinum proximity on the stabilization of the specific phases observed. The factors that limit the reversibility of the observed modifications with the different oxidation treatments are also discussed. The obtained results highlight important aspects of the cerium oxide/Pt interaction that are relevant for a complete understanding of the behavior of Pt/CeO_2_ catalysts.

## Introduction

The importance of cerium oxide in catalysis is based on its reducibility, namely on the possibility for cerium ions to reversibly switch between the 4+ and the 3+ oxidation state depending on the external conditions (Trovarelli, 1996). This property is closely linked to the material ability to reversibly store and release oxygen ions in redox reactions. In most catalytic applications cerium oxide is combined with metal nanoparticles, which complete the system functionality (Trovarelli, 1996; Cargnello et al., 2012; Trovarelli and Fornasiero, 2013). In particular, a large number of studies have demonstrated that the combination of platinum and cerium oxide allows exploiting the synergy between the two materials to obtain unprecedented activities with a minimal amount of Pt (Pierre et al., 2007; Bruix et al., 2012; Fiala et al., 2015; Jones et al., 2016). The interaction, which influences the catalytic activity, is rather complex and it involves, for example, the stabilization of ionic Pt species at specific defective ceria surface sites in catalysts with ultra-low Pt loading (Bruix et al., 2014) and a relevant charge transfer between Pt nanoparticles and cerium oxide (Lykhach et al., 2015). To identify the functionality of different atomic sites, to clarify some aspects of the interaction and to optimize the activity and/or the selectivity of catalysts, several studies made use of inverse model systems composed of well-controlled epitaxial oxide nanostructures or ultrathin films supported on metal single crystals (Suchorski et al., 2008; Senanayake et al., 2013; Grinter et al., 2016). Such systems are well-suited in particular to focus on the interplay between cerium oxide and the metal, using surface science techniques with high sensitivity and resolution.

The different properties of cerium oxide epitaxial films and nanostructures have been recently reviewed (Luches and Valeri, 2015). In particular, when Pt(111) is used as a substrate the films grow with a good epitaxial quality (Berner and Schierbaum, 2002; Grinter et al., 2010; Luches et al., 2011, 2013), with surface and subsurface oxygen vacancies (Grinter et al., 2010), step edges and domain boundaries (Grinter et al., 2010; Luches et al., 2011) as the dominant defects. The thermal stability of the oxide films indeed depends on the thickness considered, and different surface reconstructions are stabilized by thermally induced reduction (Berner and Schierbaum, 2002; Luches et al., 2014; Grinter et al., 2016). An interfacial charge transfer has been identified (Luches et al., 2015) and proposed to determine the higher reducibility of films of sub-nanometer thickness as compared to the surface of thicker deposits (Gasperi et al., 2016).

To understand the mechanisms that take place in cerium oxide during redox reactions, several recent studies have specifically focused on understanding the reduced phases stabilized on different cerium oxide surfaces (Wilkens et al., 2013a,b; Duchon et al., 2014a,b; Luches et al., 2014; Höcker et al., 2015, 2017; Grinter et al., 2016; Olbrich et al., 2017; Murgida et al., 2018). A large number of different surface reconstructions have been observed, some of which represent the truncation of a metastable bulk phase, while some others, without a bulk counterpart, are stabilized at the surface or at reduced dimensionality. The reducibility of cerium oxide, at the basis of its catalytic activity, is therefore certainly influenced by a number of factors. For example, Kozlov et al. simulated different reduced ultrathin ceria structures, and they showed that their relative stability depends on the surface lattice parameter (Kozlov et al., 2015). Indeed, epitaxial strain is only one of the parameters which may come into play, and also the electronic interaction with the substrate, the oxide surface morphology, the island size, and the temperature may play an important role (Dvorak et al., 2011; Stetsovych et al., 2013; Wilkens et al., 2013a; Luches and Valeri, 2015; Olbrich et al., 2017).

Low-energy electron microscopy (LEEM) has been shown to be able to provide useful information in this context (Flege and Grinter, 2018), allowing to access the dynamic evolution of the morphology during reduction and oxidation cycles with a lateral resolution below 10 nm and simultaneously providing structural information from electron micro-diffraction with a high spatial resolution (~250 nm) (Höcker et al., 2015, 2017; Grinter et al., 2016). Moreover, the energy-dependent electron reflectivity can provide important fingerprints of the local surface geometric and electronic properties of the investigated systems and in particular of the surface oxidation state of ceria at about 10 nm spatial resolution (Flege et al., 2013).

We report here a spatially resolved dynamic study of the formation of reduced phases at different sites in ceria/Pt systems. We identify in particular the phases which specifically originate from the direct cerium oxide—platinum interaction and the ones which originate at sites which are further from the interface. The obtained results provide a basis for a better understanding of the mechanisms that can occur in ceria-platinum catalysts, which always comprise a high density of interfacial sites.

## Materials and Methods

A Pt(111) single crystal was used as a support for the cerium oxide epitaxial nanostructures. The Pt surface was cleaned by multiple cycles of sputtering with Ar^+^ ions and annealing at 900 K in an oxygen background pressure of 5 × 10^−7^ Torr, followed by a final heating step at 1,200 K in ultra-high vacuum (UHV). Cerium was deposited by reactive molecular beam epitaxy in oxygen background pressure of 5 × 10^−7^ Torr using a home-made electron beam evaporator. The nominal thickness of deposited cerium oxide is estimated by considering the cerium flux from the evaporator and the deposition time and it is expressed in monolayer equivalents (MLE), where 1 MLE corresponds to the thickness of an O-Ce-O trilayer, 3.12 Å, fully covering the Pt surface (Sauerbrey et al., 2017). The flux of the evaporator was calibrated using our results from previous works (Luches et al., 2011), in which the thickness of a closed ceria layer on Pt(111) was estimated using a quartz microbalance coupled with scanning tunneling microscopy results. The reduction was performed using three methods, differing for the reducing agent and for the reduction temperature used: thermal treatments at 1,040 K in UHV, thermal treatments at 680 K in H_2_ background pressure of 5 × 10^−7^ Torr, and deposition of cerium at 680 K in UHV on the surface of the film. The thickness of metallic cerium is also expressed in MLE, where 1 MLE CeO_2_ ~1 MLE Ce since the atomic densities of cerium in its metal structure and in the oxide are almost the same. Reduction by cerium deposition in UHV was applied only to closed cerium oxide films, to avoid direct interactions between Ce and Pt, which are known to give origin to ordered alloyed phases (Baddeley et al., 1997; Berner and Schierbaum, 2002; Kemmer et al., 2014). The re-oxidation is performed by thermal treatments at 1,040 K in O_2_ background pressure of 1 × 10^−7^ Torr. Similar thermal treatments were shown to be effective in reducing and oxidizing cerium oxide low dimensional structures in previous studies (Duchon et al., 2014a,b; Luches et al., 2014; Höcker et al., 2015, 2017).

The morphological and structural evolution was investigated *in-situ* using a commercial Elmitec LEEM III system, equipped with multiple apertures in order to perform micro-illumination low-energy electron diffraction (μLEED) from selected areas down to ~250 nm in diameter. LEEM images and μLEED patterns were acquired in real-time during reduction and oxidation cycles. I-V LEEM curves were also acquired on selected surface areas. The μLEED and LEEM data were processed with the Gxsm software package (Zahl et al., 2010).

## Results

The dynamic modification of the structure and morphology of cerium oxide overlayers with reduction and oxidation cycles was investigated by LEEM and μLEED. I-V curves provide local information on the surface composition. Although the obtained information is only qualitative, the comparison of the curves with the ones obtained on cerium oxide samples with known stoichiometry provides relevant information on the investigated processes (Flege et al., 2013; Höcker et al., 2015). The present study focusses on two different systems: cerium oxide films completely covering the Pt substrate and well-separated cerium oxide islands with part of the substrate surface left uncovered. The continuous cerium oxide films provide essential information to interpret the more complex results obtained on cerium oxide islands. On the other hand, the islands allow for a direct observation of the interactions between the oxide and the metal at the edge sites (see, e.g., Schaefer et al., 2018).

### Continuous Cerium Oxide Films

**Figure 1:**
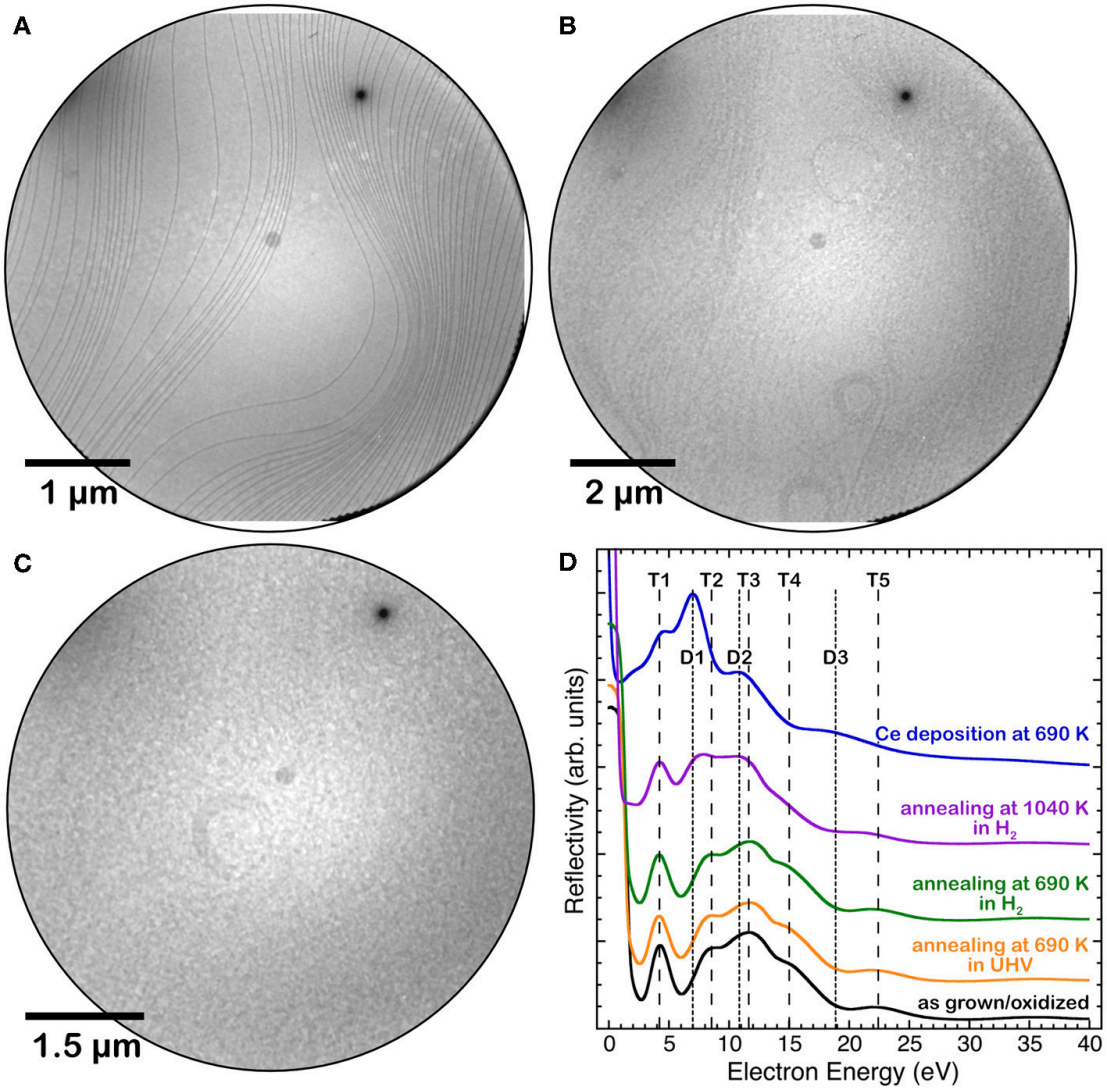
LEEM images of: **(A)** the clean Pt(111) surface, **(B)** a 5 MLE cerium oxide film grown at 720 K, **(C)** the same film after reduction in hydrogen background at 1,040 K. **(D)** I(V)-LEEM spectra of the 5 MLE film after growth at 720 K(black line) and after different reducing treatments: annealing at 690 K in UHV (yellow line), annealing at 690 K in H_2_ (green line), annealing at 1,040 K in H_2_ (purple line) and Ce deposition in UHV at 680 K on the surface (blue line).

Cerium oxide films fully covering the Pt(111) surface were obtained by depositing nominal amounts of cerium oxide above 5 MLE on the clean substrate kept at 720 K, as in Sauerbrey et al. (2017). Figure 1 shows the LEEM images of the clean Pt substrate (a) and of the film surface after the growth (b). The film morphology does not show any relevant feature within the resolution of the technique. The I-V curve acquired on the film, shown in Figure 1D (black line), has the typical shape observed for stoichiometric cerium oxide films, with a well-resolved feature at 4.5 eV (T1), three partially overlapping features at 9, 12, and 15 eV (T2–T4) and a minor feature at 22.5 eV (T5) (Flege et al., 2013; Höcker et al., 2015). Thermal treatments at 690 K in UHV or in hydrogen background pressure for 50 min did not lead to relevant modifications of the I-V curves (yellow and green lines in Figure 1D). On the contrary, a thermal treatment at 1,040 K for 185 min in hydrogen (purple line in Figure 1D) leads to significant modifications of the I-V curve, namely to a change in the energy position and relative weight of the features, which are compatible with the formation of a reduced cerium oxide phase (Flege et al., 2013; Höcker et al., 2015). A stronger reducing treatment was performed by depositing approximately 0.5 MLE of Ce in UHV on the surface at 680 K. In this case the I-V curve (blue line in Figure 1D) showed a rather marked peak at approximately 8 eV (D1), a contribution at 4.5 eV (T1) and two minor features at 11 and 19 eV (D2, D3), which are well compatible with the formation of a fully reduced Ce_2_O_3_ bixbyite phase (Flege et al., 2013; Höcker et al., 2015). The LEEM image acquired after reduction in hydrogen background at 1,040 K (Figure 1C) does not show pronounced morphological modifications compared to the image acquired before oxidation (Figure 1B).

**Figure 2:**
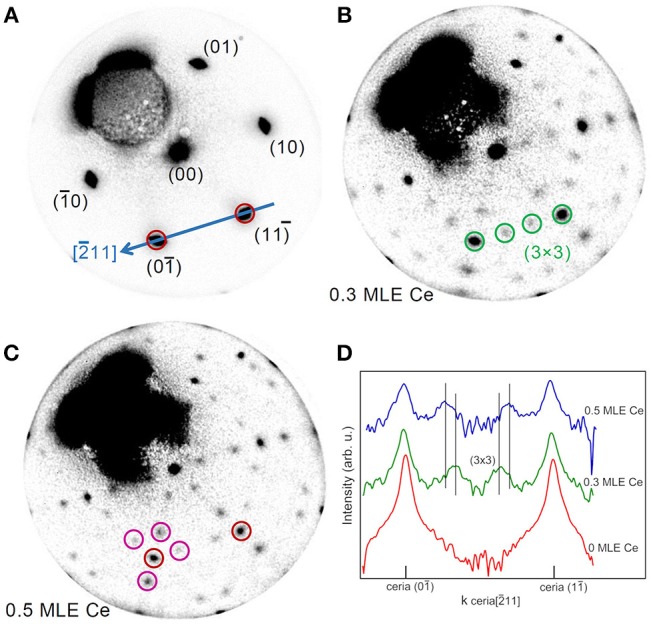
Time-lapse LEED patterns sequence acquired at *E* = 40.0 eV during reduction of a 5 MLE cerium oxide film by Ce deposition at 680 K on the oxide film surface: **(A)** as prepared (0.0 ML Ce); **(B)** after 3,600 s (0.3 ML Ce); **(C)** after 6,000 s of deposition (0.5 ML Ce). **(D)** Profiles of the LEED patterns in **A**, **B**, and **C** along the cerium oxide [101] direction (blue arrow in **A**).

The evolution of the structure with reducing treatments was followed by LEED. Figure 2 shows selected patterns acquired during Ce deposition on the film surface, while the full LEED movie is included as Supplementary Video 1. Before the treatment the LEED shows only the cerium oxide related spots and no substrate related spot (Figure 2A), which indicates that the substrate is fully covered by an epitaxial film with a good crystalline quality. After the deposition of 0.3 MLE of Ce a clear (3 × 3) superstructure appears (Figure 2B). This surface periodicity in reduced cerium oxide films was already observed to be formed after Ce deposition on the surface of a 3 nm thick cerium oxide films on Cu(111) followed by annealing at 870 K by Duchon et al. and it was ascribed to the CeO_1.67_ phase with a specific surface structure (Duchon et al., 2014b). Subsequently, the same phase was observed to appear also on ceria films grown on Ru(0001) using thermal reducing treatments (Duchon et al., 2014a; Höcker et al., 2015). By depositing larger amounts of metallic cerium the superstructure-related spots gradually move toward the cerium oxide related spots, reaching a periodicity of approximately (3.5 × 3.5) after the deposition of 0.5 MLE of Ce. The LEED intensity profiles along the [101] direction, reported in Figure 2D, show the shift of the (3 × 3)-related spots toward the (11) and (10) ceria spots. A previous work by Höcker et al. (2015) demonstrated that if two coexisting phases with slightly different surface periodicities (e.g., 3 × 3 and 4 × 4) gradually transform from one into the other in a non-correlated way, the lattice parameter of the resulting periodicity in LEED patterns progressively changes between the two values determined by the individual phases (Höcker et al., 2015). It seems therefore reasonable to assume that the (3.5 × 3.5) periodicity originates from an incomplete transformation of the (3 × 3) phase into a phase with a (4 × 4) periodicity. The latter corresponds to the bixbyite Ce_2_O_3_ phase, which was observed to be stabilized by reduction of ceria films on Cu(111) (Duchon et al., 2014b) and on Ru(0001) (Duchon et al., 2014a; Höcker et al., 2015).

**Figure 3:**
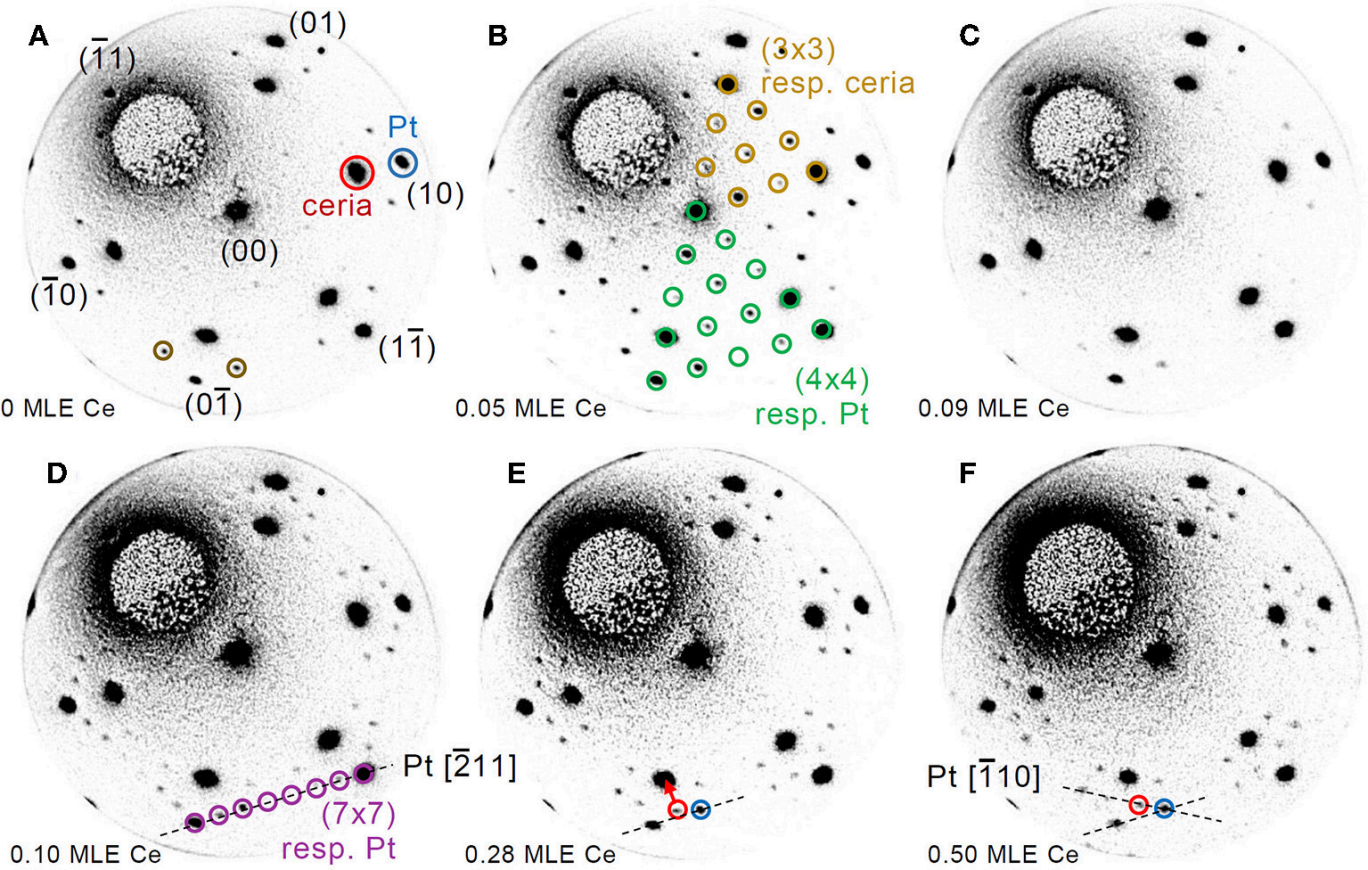
Time-lapse LEED pattern sequence acquired at *E* = 40.0 eV during reduction of a 1.5 MLE cerium oxide film by Ce deposition at 680 K on the oxide film surface: **(A)** as prepared (0.00 ML Ce); **(B)** after 245 s (0.05 ML Ce); **(C)** after 435 s (0.09 ML Ce); **(D)** after 540 s (0.10 ML Ce); **(E)** after 1,425 s (0.28 ML Ce); **(F)** after 2,495 s of deposition (0.50 ML Ce).

In order to investigate the effect of interface proximity on the reduced phases that are stabilized, reduction was induced by cerium deposition on the surface of a much thinner film of 1.5 MLE. Figure 3 reports a time-lapse LEED pattern sequence during the deposition of selected amounts of cerium, while the full LEED movie is included as Supplementary Video 2. Before the treatment (Figure 3A) the LEED pattern shows both the spots related to cerium oxide and those related to the Pt(111) surface plus some extra spots (brown circles in Figure 3A), probably originating from a non-complete oxidation of the cerium oxide film. After the deposition of ~0.05 MLE of Ce a well-ordered (3 × 3) phase appears (Figure 3B), in analogy with the phase obtained on thicker continuous films after the deposition of 0.3 MLE of Ce (Figure 2B). After the deposition of 0.09 MLE of cerium (Figure 3C) the (3 × 3) phase disappears and the surface shows a pattern similar to the one observed before reduction (Figure 3A), which might be due to random creation of further oxygen vacancies. With further reduction a phase with a (7 × 7) periodicity with respect to the Pt surface becomes clearly visible, which corresponds to an approximate (5 × 5) periodicity with respect to cerium oxide (Figure 3D). As the reduction proceeds (Figures 3E,F) some of the superstructure spots gradually move toward the main cerium oxide spots finally forming a 5/2(√3 × √3)R30°reconstruction after the deposition of ~0.5 MLE of Ce. Also in this case the gradual movement of some of the LEED spots is interpreted as due to two kinds of spatially uncorrelated domains with the (5 × 5) and the 5/2(√3 × √3)R30° reconstruction which gradually transform one into the other, as in Höcker et al. (2015).

### Cerium Oxide Islands

**Figure 4:**
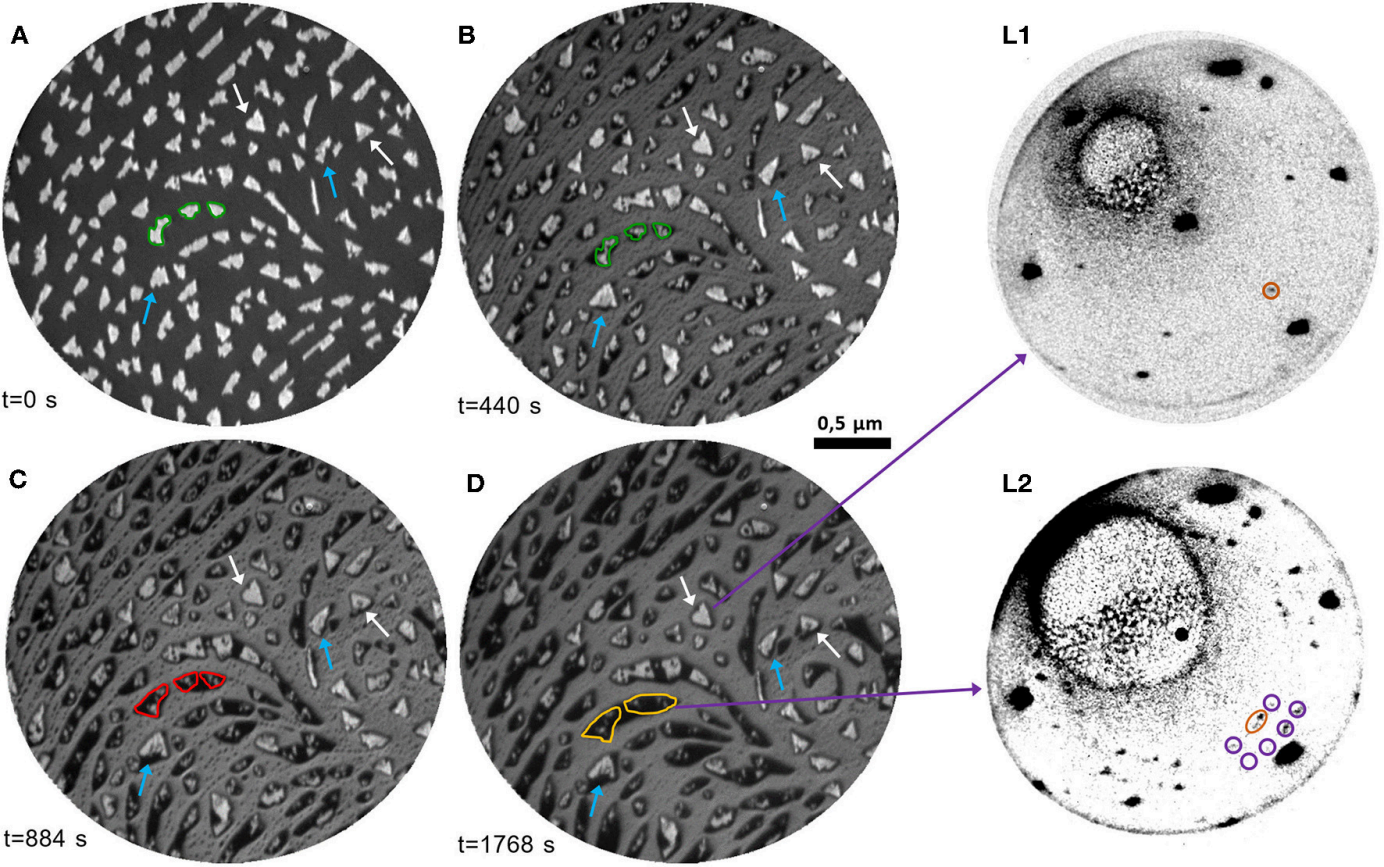
**(A–D)** Time-lapse LEEM image sequence at *E* = 12.0 eV acquired during thermal reduction in UHV at 1,040 K of cerium oxide islands of nanometric thickness (1.5 MLE): **(A)** before the reduction; **(B)** after 440 s; **(C)** after 884 s; **(D)** after 1,768 s. The edge of selected islands is highlighted in green in **(A)** and reported in **(B)**, to emphasize the decrease of the bright areas and the increase of the dark areas. The expansion of the low-reflectivity phase in the same area with further reduction is highlighted in red in **(C)**, and in yellow in **(D)**. The blue arrows indicate bright islands which become wider and more regular during the first stages of the treatment. The white arrows point at regular triangular islands which undergo only a decrease in reflectivity with the treatment. (L1)-(L2): μLEED patterns (*E* = 40.0 eV) acquired on selected areas of **(D)**: (L1) on a bright triangular island; (L2) on a low-reflectivity area.

To have more detailed information on the interaction between cerium oxide and Pt, we investigated the processes occurring with thermal reduction on a sample obtained by growing 1.5 MLE cerium oxide on the Pt surface at 1,040 K to allow for the formation of well-separated cerium oxide islands, using the procedures identified in one of our previous studies (Sauerbrey et al., 2017). Figure 4A reports a typical LEEM image of the sample surface. The bright features can be ascribed to cerium oxide, which has a higher reflectivity than the bare Pt surface at 12 eV (see Figure 1D and Sauerbrey et al., 2017). The lateral size of the ceria features varies between ~50 and 200 nm and their shape appears mainly irregular, being partially constrained by the substrate step edges. The islands that nucleate on flat areas of the Pt substrate, far from step edges, have a more regular triangular shape as they have grown from a single or only very few nuclei (highlighted by white arrows in Figure 4). The average height of the islands, estimated using the Pt surface coverage (25 ± 1%) and the cerium oxide equivalent thickness, amounts to approximately 6 ML.

The evolution of the morphology of the sample during thermal treatments at 1,040 K in UHV was followed by LEEM in real-time (see also Supplementary Video 3). Figures 4B–D represent selected LEEM images during reduction. The image acquired after 440 s (Figure 4B) evidences significant morphological modifications of the sample. Most of the islands are surrounded by an area characterized by a very low reflectivity, which appears darker than the substrate. The low-reflectivity phase partially extends also along the step edges, partially decorating them with an almost continuous profile. The extent of the bright part of the islands decreases with reduction, as shown for example by comparing the areas highlighted in green in Figures 4A,B. Simultaneously, some of the bright islands increase in size and acquire a more regular triangular shape (blue arrows). With longer times, the low-reflectivity phase surrounding the ceria structures further extends on the substrate surface, while the bright part of the islands decreases in size (areas highlighted in red and yellow in Figures 4C,D). The morphology and contrast of the larger triangular islands (white arrows) are almost unchanged, except for a mild decrease in reflectivity. The latter can be rationalized by considering the modifications of the I-V curves induced by the different reduction treatments in Figure 1D. The I-V curve T3 peak at 11.6 eV observed in stoichiometric cerium oxide film does not change significantly in intensity after mild reduction treatments, while a pronounced decrease in reflectivity at ~12 eV is observed after strong reduction. We therefore interpret the mild decrease of reflectivity of the ceria islands as originating from an intermediate reduction, in accordance with previous findings from spatially resolved I(V) analysis for chemical reduction of cerium oxide islands on Ru(0001) (Kozlov et al., 2015). The nature of the phase with a lower reflectivity than Pt at 12 eV is instead less clear, although a similar behavior was observed also on reduced ceria films on a Ru(0001) support (Höcker et al., 2017).

In order to have more information on the nature of the different phases obtained with increasing reduction μLEED images were acquired on selected areas of the fully reduced sample. Regions including mainly the brighter islands (Figure 4 L1) show dominant spots with hexagonal symmetry ascribed to the Pt(111) surface, together with a pattern of lower intensity and smaller size in reciprocal space, compatible with a reduced cerium oxide phase without specific ordering of oxygen vacancies, considering the decreased reflectivity compared to fully oxidized phases (Figures 4A,C). The cerium oxide related spots are 5° rotated with respect to the Pt ones, possibly due to the higher stability of the rotated orientation (Luches et al., 2011; Sauerbrey et al., 2017). On the contrary, on the areas comprising mainly the phase with a reflectivity lower than Pt μLEED shows superstructures with a 5/2(√3 × √3)R30° periodicity (Figure 4 L2). In the literature a similar superstructure was observed by LEED at the early stages of Pt-Ce alloy formation and ascribed to a metallic Pt_5_Ce alloy (Baddeley et al., 1997; Kemmer et al., 2014). This suggests that there might be a non-negligible tendency for interatomic exchange between cerium oxide and Pt induced by the thermal reduction.

**Figure 5:**
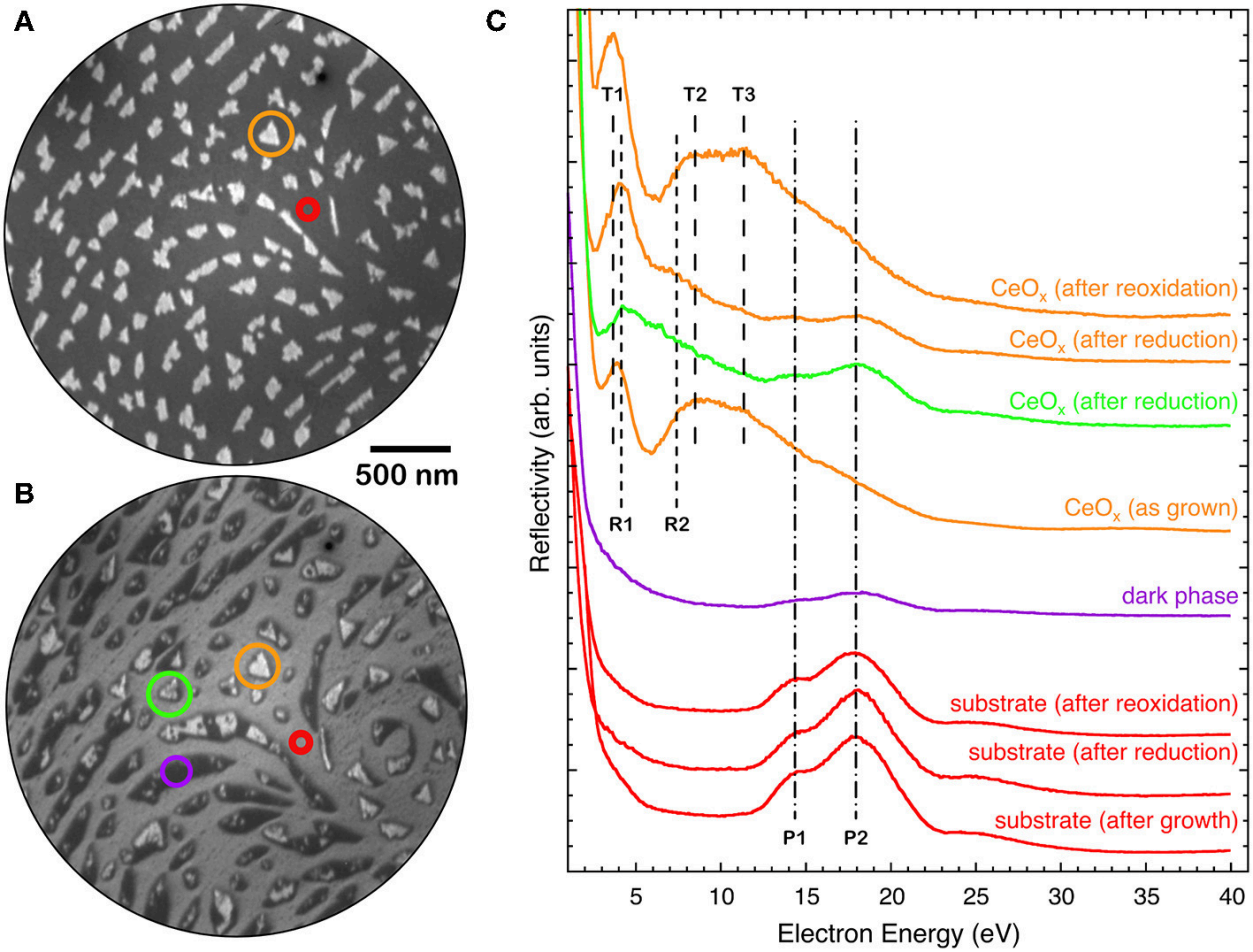
LEEM image (*E* = 12.0 eV) of a 1.5 MLE ceria film **(A)** as prepared and **(B)** after reduction [same as (Figures 4A,D)]. **(C)** I-V LEEM curves acquired on selected areas of the as-prepared, reduced and re-oxidized samples: triangular island (orange), substrate (red), low-reflectivity (dark) phase (purple), and island with decreased reflectivity (green).

Figure 5 reports the I-V curves in selected areas of the 1.5 MLE sample as-prepared, after reduction and after re-oxidation. The spectra obtained on areas between the cerium oxide islands correspond to the one expected for clean platinum, with features at 14 eV (P1) and at 18 eV (P2) (red curve in Figure 5C). The as-grown cerium oxide islands exhibit an I-V curve that contains both the cerium oxide related features at 3.5, 8, and 11 eV, T1-T3 in analogy with Figure 1, and a weak Pt-related P2 feature at 18 eV (yellow curve in Figure 5C). After reduction the I-V curve on the bare substrate areas is not significantly altered (red curve in Figure 5C), apart from an overall increase of intensity possibly originating from the desorption of oxygen from the Pt surface. The dark areas have a reflectivity that is lower than that of the Pt at all energies (purple curve in Figure 5C). The islands, which still appear bright after reduction, have an I-V curve with a dominant R1 peak at low energies around 4 eV, slightly shifted to higher energies compared to the T1 peak before reduction (yellow curve in Figure 5C). A broad feature, R2, appears at 7 eV together with attenuated Pt-related P1 and P2 features at 14 and 18 eV. The regular islands which show a mild reflectivity decrease at ~12 eV with the reducing treatment, like the one highlighted in green in Figure 4B, have an I-V curve which appears somewhat intermediate between the ones observed on bright islands and on the low-reflectivity phase (green curve in Figure 5C).

**Figure 6:**
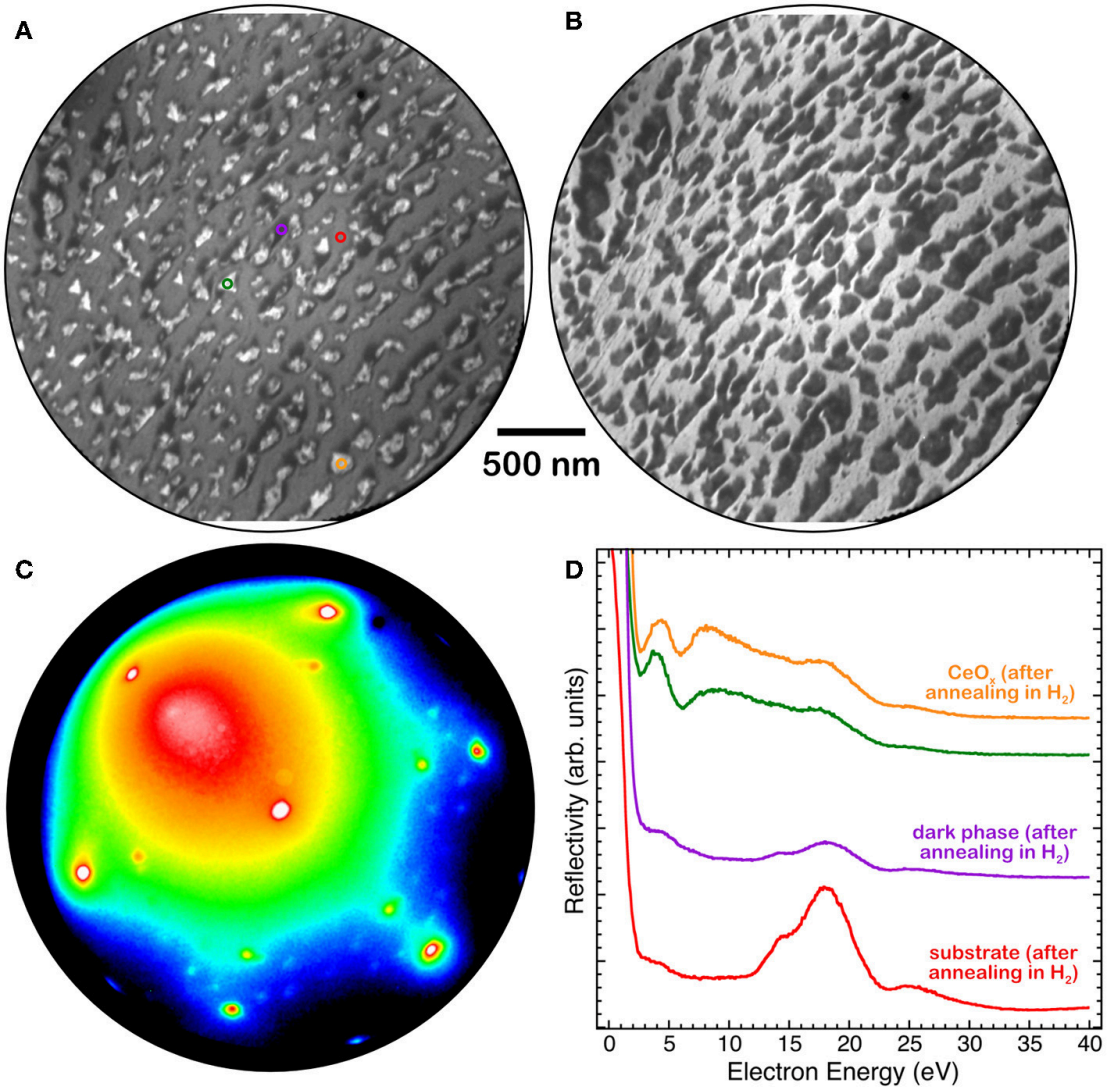
**(A)** LEEM image at *E* = 12 eV, **(B)** LEEM image at *E* = 17 eV, **(C)** LEED pattern at *E* = 40 eV and **(D)** I-V LEEM curves, acquired on selected areas of a 1.8 MLE cerium oxide film grown at 1,040 K and reduced by H_2_ exposure (P_H2_ = 1 × 10^−7^ Torr) at 680 K: triangular island (orange and green), substrate (red), low-reflectivity (dark) phase (purple).

A very similar phase with low reflectivity surrounding the brighter islands was also observed after exposure of the ceria islands to hydrogen at a pressure of 1 × 10^−7^ Torr at 680 K (Figures 6A,B). The I-V curves of the different regions, bright islands, dark island areas and substrate (Figure 6D), are compatible with the ones observed after the thermal treatment in UHV (Figure 5). The LEED pattern acquired on the low reflectivity areas (Figure 6C) shows a reconstruction very similar to the one observed after reduction by thermal treatments in UHV (Figure 4 L2).

**Figure 7:**
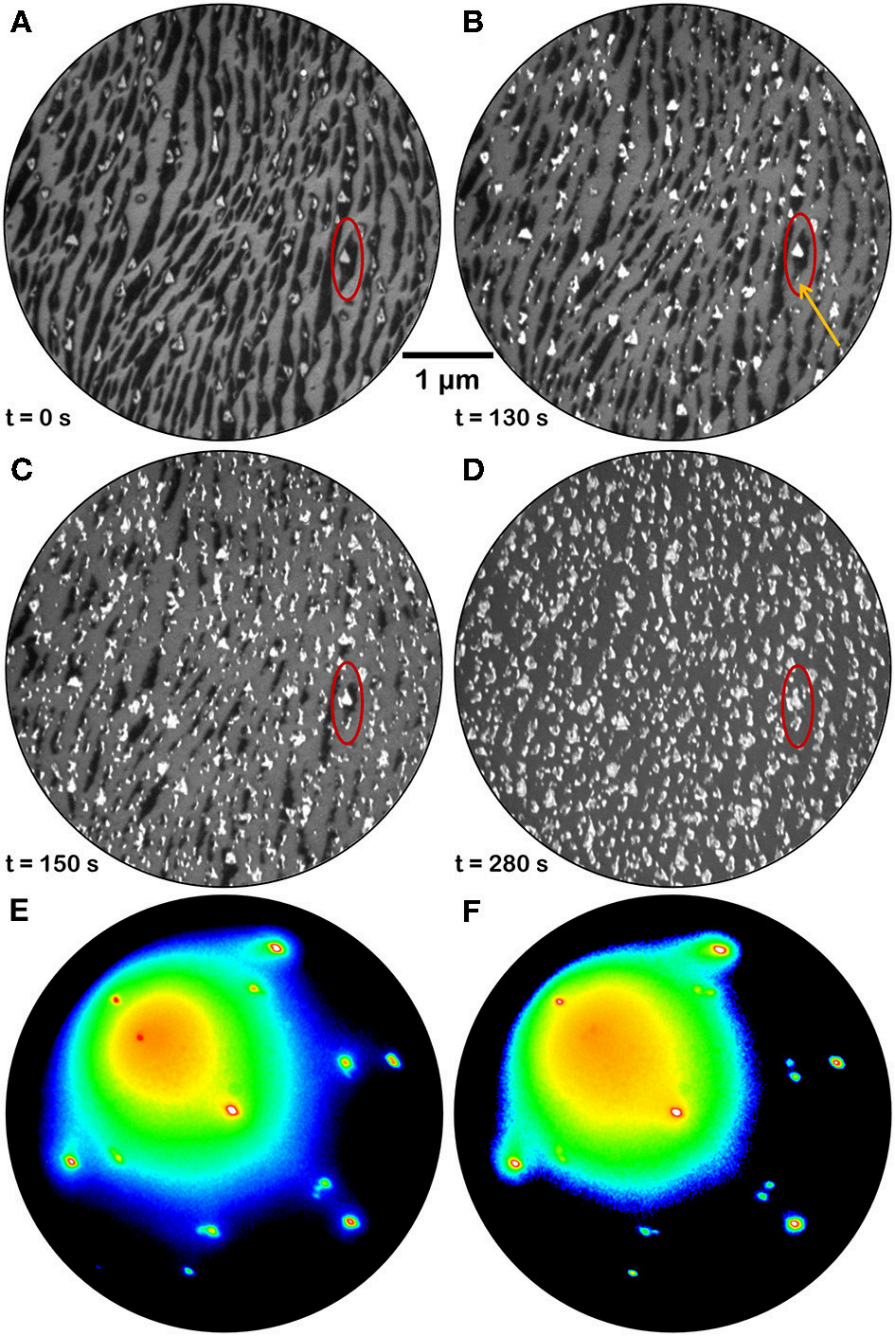
Time-lapse LEEM image sequence at *E* = 12.0 eV acquired during re-oxidation of a fully reduced ceria sample (1.5 MLE) by annealing in O_2_ background pressure (*P* = 1 × 10^−7^ Torr) at 1,040 K: **(A)** before the re-oxidation; **(B)** after 130 s; **(C)** after 150 s; **(D)** after 280 s. The red ellipse allows to follow the evolution of the morphology in a selected surface region. The orange arrow in **(B)** evidences a bright feature that was absent before the treatment and that is growing with time **(C,D)**. **(E,F)** μLEED patterns (*E* = 40 eV) acquired on two islands of different shape after full reduction and subsequent re-oxidation by exposure to oxygen at P_O2_ = 1 × 10^−7^ Torr for 5 min at 1,040 K.

The evolution of the morphology of fully reduced cerium oxide islands during oxidation in oxygen background pressure at 1,040 K was also investigated. Figure 7 reports a time-lapse sequence of LEEM images acquired during the treatment (see also Supplementary Video 4). Already after the first few minutes of oxygen exposure the low-reflectivity phase, initially covering a large part of the surface, starts to vanish while the bright areas increase in size and their reflectivity is increased (area highlighted in red in Figure 7). New bright islands also appear during the treatment, like for example the one indicated with a yellow arrow in Figure 7B. With further oxygen exposure the low-reflectivity phase completely disappears, and it is replaced by bright islands with a non-uniform contrast and an irregular shape. The LEED patterns after re-oxidation are very close to the ones acquired before reduction, demonstrating the reversibility of the structure (Figures 7E,F). In analogy, also the I-V curves acquired before reduction and after re-oxidation appear very close, demonstrating the reversibility of the surface stoichiometry, while the I-V curves on bare Pt surface areas appear unaltered (Figure 5C).

## Discussion

The present study points at understanding the dynamics of the processes which occur at the interface between ceria and platinum during redox processes.

The main result of the work is the evidence for a strong interaction between the two materials involving non-negligible mass transport already at the early stages of isothermal reduction starting at the cerium oxide island edge sites (Figure 3). At these sites the formation of oxygen vacancies is likely favored by reduced coordination and by the proximity of Pt which possibly induces a decrease in the oxygen vacancy formation energy by charge transfer (Luches et al., 2015). At the earlier reduction stage the stability of cerium oxide islands with irregular shape is significantly lower than the one of islands with regular triangular shape (Figure 4), probably because the reduction is more pronounced where the island edges are less regular, which typically implies the presence of kinks and other kinds of defects, and a low structural order in the long range. The thicker and more regular islands are not significantly altered at the investigated temperature at this earlier stage. Some of the thicker islands increase their lateral size at this stage because some of the neighboring islands with smaller lateral size get some mobility and possibly merge with them. At a later stage most of the islands are partially converted into the alloy and their lateral size is decreased. The low-reflectivity phase, which appears and expands with reducing treatments, is ascribed to a surface alloy based on its LEED pattern although it has a reflectivity which is very low at all energies, as expected for a rough surface with a low crystal quality (Sauerbrey et al., 2016). This may indicate that the surface mobility at the used temperature (1,040 K) is not high temperature (1,040 K) is not high enough to reach a good surface ordering, although the long-range ordered Ce-Pt surface alloy. We note that the appearance of a low-reflectivity phase with reduction low-reflectivity phase with reduction was observed also on the Ru(0001) surface, although it did not show any diffraction pattern (Höcker et al., 2017), probably due to the different miscibility of cerium and Ru, given the alloys (Colinet, 1995). A phase with the same surface periodicity (and LEED dynamic) is observed also on a 1.5 MLE cerium oxide films fully covering the Pt surface on which the reduction is induced by cerium deposition at 680 K (Figure 3F), and also after reduction of a 1.8 MLE film (grown at 1,040 K) induced by H_2_ reduction of a 1.8 MLE film (grown at 1,040 K) induced by H_2_ exposure of at 680 K (Figure 6). The formation of a surface alloy on thermally reduced ceria films on Pt(111) was also hypothesized based on atomic and Kelvin probe force microscopy experiments (Gasperi et al., 2018). These evidences indicate that surface alloy formation is a process which also occurs at intermediate temperatures in reducing conditions.

An important aspect is that the extended low reflectivity phase can be transformed back to confined cerium oxide islands by oxidation at elevated temperature (Figure 7). The partially reduced bright islands increase in size, suggesting that re-oxidation is more favorable at the step edges between reduced ceria and the Pt-Ce alloy, inducing a preferential oxide formation at the sides of the existing islands accompanied by a progressive shrinking of the low-reflectivity phase. Simultaneously also the reflectivity at 12 eV of partially reduced cerium oxide islands increases, evidencing a change in oxidation state and suggesting a filling of surface oxygen vacancies. Moreover, new bright islands appear in isolated sites of the low reflectivity phase, possibly where some cerium oxide nanostructures below the LEEM resolution were left by the reduction treatment or at some other defect sites created during alloy formation. The resulting different morphology of the ceria islands after re-oxidation is probably due to kinetic barriers which limit the diffusion and originate less regular structures, compared to the ones formed when Ce adatoms are co-deposited with O_2_ on the clean, very smooth Pt surface. At the investigated oxygen pressures and temperatures, both the closed films and the ceria islands were fully re-oxidized in relatively short times, of the order of tens of minutes. Indeed, it would be interesting to follow also the dynamics of re-oxidation in different conditions. The efficiency for molecular oxygen dissociation on the Pt surface for example is expected to contribute to the kinetics of re-oxidation at milder oxidizing conditions. We suggest this as a relevant issue to be investigated in future studies, to obtain a complete picture of the redox process. Indeed, a non-negligible and reversible interatomic exchange is likely to occur also in Pt-ceria real catalysts during redox reactions, likely on a shorter length scale determined by the working temperature and by pressure of the reactant. The process needs to be considered in studies devoted to the optimization of cerium oxide/Pt catalysts, for example by minimizing or substituting platinum.

**Figure 8:**
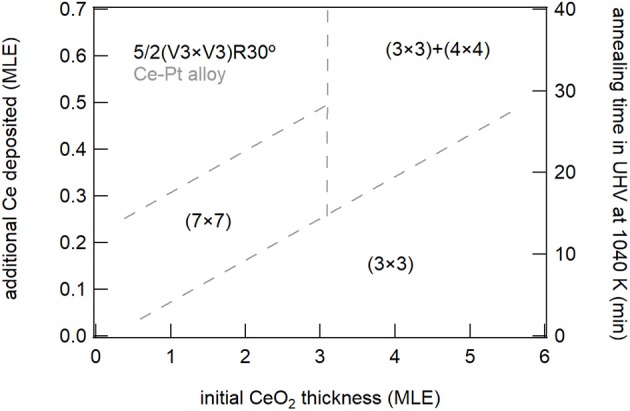
Schematic phase diagram summarizing the different reduced phases observed on the surface of ceria films for the different nominal thicknesses and in the different reducing conditions.

The present work also allows to draw some important considerations on the factors which induce the stabilization of specific reduced phases in cerium oxide. A phase diagram summarizing the phases observed for the different ceria film thicknesses and at the different reducing conditions is shown in Figure 8. The (3 × 3) phase was observed both on 1.5 MLE and on 5 MLE films, although in the first case a much smaller amount of cerium was required for its stabilization. The Pt substrate possibly favors the formation of the (3 × 3) phase which has a surface size compatible with the coincidence cell at the ultrathin limit (Luches et al., 2013). The CeO_1.67_ (3 × 3) phase was already observed in cerium oxide ultrathin films supported on different metal substrates, e.g., Cu(111) (Duchon et al., 2014b; Höcker et al., 2015), while it was not observed on bulk surfaces (Olbrich et al., 2017), although a recent theoretical study showed that it can result from the (111) termination of a metastable bulk Ce_3_O_5_ phase (Murgida et al., 2018). The reduced dimensionality in the out of plane direction probably plays a role in its stabilization, given the higher structural flexibility of thin films compared to bulk surfaces. The Ce_2_O_3_ (4 × 4) phase was shown to coexist with the (3 × 3) phase on 5 ML films at the maximum degree of reduction accessed, while on thinner films it was not observed, being probably less stable than other reduced phases. The phase was also observed in previous studies on ultrathin films on different substrates (Duchon et al., 2014a,b; Höcker et al., 2015), as well as on bulk-like surfaces (Olbrich et al., 2017). The reduced (5 × 5) phase, observed in this work on 1.5 MLE films at intermediate reduction (Figure 3D), was never observed in previous studies. We speculate that its stabilization may also be ascribed to epitaxy with the Pt substrate, since a 5:7 coincidence between cerium oxide and platinum is possible and it implies a slight surface expansion of the cerium oxide surface lattice (Luches et al., 2013), indeed favored by reduction since Ce^3+^ ions have a larger radius than Ce^4+^ ions. The (5 × 5) phase gradually evolves with further reduction toward the 5/2(√3 × √3)R30° phase which is related to the initial stages of an alloy formation as discussed above. The alloy was observed at low thickness and moderate degrees of reduction, while for thicker films the onset for alloy formation was found at higher degrees of reduction. The plethora of surface reconstructions, the fast transformation between the different phases and the partially different behavior of cerium oxide layers close to the interface compared to the surface of thin films highlight the complexity of the processes that occur at the interface between these two technologically important materials during redox processes.

Finally, this work proves that for a thorough understanding of the complex phenomena which occur during reduction and oxidation a real-time investigation not only of the evolution in morphology, but also of the structure and electronic properties is extremely important to fully understand the system behavior.

## Conclusions

We have investigated the dynamic changes in morphology, structure, and electronic properties in cerium oxide films and confined islands with reducing and oxidizing treatments. We relate the observed modifications to the formation of different reduced structural phases, and we discuss the effect of temperature, of dimensionality and of the interaction with platinum for their stabilization. In particular, the CeO_1.67_ (3 × 3) phase gradually transforms into the Ce_2_O_3_ (4 × 4) bixbyite phase, with increasing reduction in sites which are not in close proximity with the Pt interface. This is in contrast to the very early stage of the reduction process, more prominent where the lateral oxide-metal interface is less smooth and more defective. The resulting (3 × 3) phase is stabilized at milder conditions in ultrathin films due to an important role of the Pt substrate. At higher degrees of reduction the interaction between cerium oxide and Pt induces the formation of more complex phases, and it finally leads to a partial interdiffusion between substrate and overlayer atoms. The structural modifications are reversible with oxidizing treatments, whereas a complete restoration of the original morphology is partially hindered by kinetic barriers.

## Author Contributions

PL, JIF, JF, and SV conceived and designed the experiment. GG and MS did the sample growth, the measurements and the data analysis. JIF and JF supervised the experiment and the data analysis. PL wrote the manuscript draft. All authors contributed to data interpretation, discussion of results and manuscript critical revisions.

### Conflict of Interest Statement

The authors declare that the research was conducted in the absence of any commercial or financial relationships that could be construed as a potential conflict of interest.

## Supplementary Material

Supplementary Video 1 LEED movie (*E* = 40 eV) during reduction of a 5 ML cerium oxide film by Ce deposition at 680 K on the film surface.

Supplementary Video 2 LEED movie (*E* = 40 eV) during reduction of a 1.5 ML cerium oxide film by Ce deposition at 680 K on the film surface.

Supplementary Video 3 LEEM movie (*E* = 12 eV) of cerium oxide islands of nanometric thickness during thermal reduction in UHV at 1,040 K.

Supplementary Video 4 LEEM movie (*E* = 12 eV) of reduced cerium oxide islands of nanometric thickness during thermal reoxidation at 1,040 K in oxygen background pressure (P_O2_ = 1 × 10^−7^ Torr).
